# *IRAV* (*FLJ11286*), an Interferon-Stimulated Gene with Antiviral Activity against Dengue Virus, Interacts with MOV10

**DOI:** 10.1128/JVI.01606-16

**Published:** 2017-02-14

**Authors:** Corey A. Balinsky, Hana Schmeisser, Alexandra I. Wells, Sundar Ganesan, Tengchuan Jin, Kavita Singh, Kathryn C. Zoon

**Affiliations:** a Cytokine Biology Section, National Institute of Allergy and Infectious Diseases, National Institutes of Health, Bethesda, Maryland, USA; b Biological Imaging Section, Research Technologies Branch, National Institute of Allergy and Infectious Diseases, National Institutes of Health, Bethesda, Maryland, USA; c Structural Immunobiology Unit, Laboratory of Immunology, National Institute of Allergy and Infectious Diseases, National Institutes of Health, Bethesda, Maryland, USA; d Structural Biology Section, Research Technologies Branch, National Institute of Allergy and Infectious Diseases, National Institutes of Health, Bethesda, Maryland, USA; Washington University School of Medicine

**Keywords:** IRAV, FLJ11286, C19orf66, UPF0515, dengue, flavivirus, interferon, MOV10, P body

## Abstract

Dengue virus (DENV) is a member of the genus Flavivirus and can cause severe febrile illness. Here, we show that FLJ11286, which we refer to as IRAV, is induced by DENV in an interferon-dependent manner, displays antiviral activity against DENV, and localizes to the DENV replication complex. IRAV is an RNA binding protein and localizes to cytoplasmic processing bodies (P bodies) in uninfected cells, where it interacts with the MOV10 RISC complex RNA helicase, suggesting a role for IRAV in the processing of viral RNA. After DENV infection, IRAV, along with MOV10 and Xrn1, localizes to the DENV replication complex and associates with DENV proteins. Depletion of IRAV or MOV10 results in an increase in viral RNA. These data serve to characterize an interferon-stimulated gene with antiviral activity against DENV, as well as to propose a mechanism of activity involving the processing of viral RNA.

**IMPORTANCE** Dengue virus, a member of the family Flaviviridae, can result in a life-threatening illness and has a significant impact on global health. Dengue virus has been shown to be particularly sensitive to the effects of type I interferon; however, little is known about the mechanisms by which interferon-stimulated genes function to inhibit viral replication. A better understanding of the interferon-mediated antiviral response to dengue virus may aid in the development of novel therapeutics. Here, we examine the influence of the interferon-stimulated gene *IRAV* (*FLJ11286*) on dengue virus replication. We show that IRAV associates with P bodies in uninfected cells and with the dengue virus replication complex after infection. IRAV also interacts with MOV10, depletion of which is associated with increased viral replication. Our results provide insight into a newly identified antiviral gene, as well as broadening our understanding of the innate immune response to dengue virus infection.

## INTRODUCTION

Dengue virus (DENV), a member of the genus Flavivirus, is a positive-sense RNA virus comprised of a nucleoprotein core surrounded by a host-derived membrane. DENV is transmitted through the bite of a mosquito vector and is the etiologic agent of a spectrum of illnesses that can range from mild fever to the potentially fatal dengue hemorrhagic fever/dengue shock syndrome. Due to geographic expansion of its insect vector, as well as increased travel and urbanization, dengue virus is of increasing importance to public health. Dengue virus infects an estimated 390 million people each year, with more than 2.5 billion people living in regions where they are at risk of dengue virus transmission (1–4).

Activation of interferon (IFN) signaling pathways results in the upregulation of hundreds of interferon-stimulated genes (ISGs) (5–7). Interferon-stimulated genes encoding viperin, TRAIL, IFITM1, IFITM2, IFITM3, ISG20, and TRIM56 have all been shown to inhibit DENV replication through a variety of mechanisms (8–12). In addition, a number of ISGs, including IFIT2, IFIT1, ISG15, TRIM22, and TRIM79α genes, have been found to have activity against other flaviviruses (13–18). High-throughput screens have implicated a number of other ISGs in inhibition of flavivirus replication (6, 19).

Previously, we identified *FLJ11286* as one of a number of genes that were upregulated in Daudi cells in response to treatment with IFN (20) and showed that they displayed antiviral activity against encephalomyocarditis virus (EMCV) (21). FLJ11286 has also been shown by our laboratory and others to have antiviral activity against DENV (22, 23). *FLJ11286*, which we refer to here as *IRAV* (*interferon-regulated antiviral gene*) (also annotated as *C19orf66*, *UPF0515*, or *RyDEN*), encodes a protein 291 amino acids (aa) in length with a calculated molecular mass of 33.1 kDa. Analysis of published microarray data suggests that *IRAV* (*FLJ11286*) is upregulated in response to type I and type II IFNs (6, 20, 24–26). *IRAV* (*FLJ11286*) has been shown in microarray screens to be upregulated in response to the yellow fever virus vaccine (27), as well as after infection with a number of different pathogens, including adenovirus (28), influenza virus (29), Lassa virus (30), and ebola and Marburg viruses (31), as well as after infection with human herpesvirus 8 and human herpesvirus 1 (32, 33). In addition, proteomic analysis of the hepatitis C virus (HCV) interactome identified IRAV (FLJ11286) as one of 214 human proteins interacting with the HCV NS3 protein (34), suggesting a role for IRAV in the host pathogen response.

Here, we show that IRAV is upregulated in response to DENV infection in an IFN-dependent manner. Upregulation of IRAV in response to IFN-β treatment can be blocked by disrupting the canonical ISGF3 pathway. CRISPR/Cas9-mediated knockout of IRAV resulted in increased titers of DENV, as well as of EMCV. We also demonstrate that IRAV associates with DENV proteins and localizes to the viral replication complex. IRAV is an RNA binding protein and localizes to P bodies in uninfected cells. IRAV also associates with the host RNA binding proteins UPF1 and HuR (ELAV1) and interacts with MOV10 (a RISC complex RNA helicase), suggesting a role for IRAV in processing or stability of RNA. Furthermore, we propose a mechanism of action for IRAV that utilizes intrinsic RNA decay pathways. These pathways have been shown to be of increasing importance to the life cycles of multiple viruses, as well as in an array of cellular processes.

## RESULTS

### IRAV is upregulated after dengue virus infection in an interferon-dependent manner.

**FIG 1:**
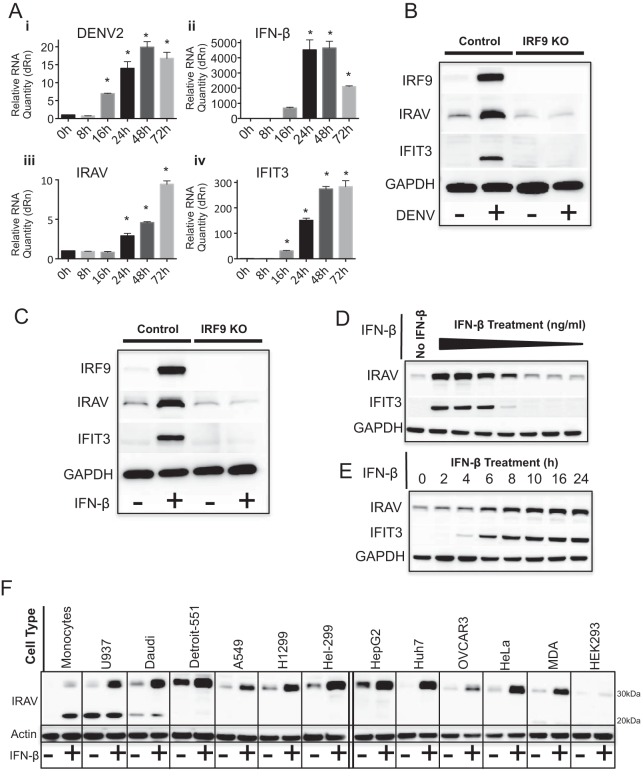
*IRAV* is an interferon-stimulated gene. (A) qRT-PCR analysis of RNA expression in A549 cells after DENV infection at 0, 8, 16, 24, 48, and 72 h postinfection. Cells were infected with DENV at an MOI of 0.1 for 1 h, and samples were collected at time zero and the indicated time points. The samples were analyzed by qRT-PCR for DENV RNA (i), as well as for expression of *IFN*-β (ii), *IRAV* (iii), and the ISG *IFIT3* (iv). The samples were normalized to the *HPRT* housekeeping gene, and the change in expression was calculated relative to time zero. The error bars represent standard deviations. *, *P* < 0.05. (B) Western blot analysis of IRAV expression in control A549 cells or IRF9 KO cells after DENV infection (72 h). Uninfected (−) and DENV-infected (+) A549 or IRF9 KO cells were analyzed by Western blotting for IRF9, IRAV, or IFIT3. GAPDH was used as a loading control. (C) Western blot analysis of IRAV expression in control A549 or IRF9 KO cells after treatment with IFN-β. The cells were either left untreated (−) or treated with IFN-β (+) for 16 h, followed by Western blot analysis for expression of IRF9, IRAV, or IFIT3. GAPDH was used as a loading control. (D) Western blot analysis of IRAV expression in HeLa cells after treatment with 10-fold dilutions of IFN-β (30 to 0.00003 ng/ml) for 16 h. Samples were examined by Western blotting for expression of IRAV and IFIT3. GAPDH was used as a loading control. (E) Western blot analysis of IRAV expression in HeLa cells after treatment with IFN-β (3 ng/ml) at the indicated time points. Samples were examined by Western blotting for expression of IRAV and IFIT3. GAPDH was used as a loading control. (F) Western blot analysis of IRAV expression in various cell lines. The indicated cell lines were either left untreated (−) or treated with IFN-β (+) (10 ng/ml) for 16 h, followed by Western blot analysis for IRAV. Actin was used as a loading control.

To determine if IRAV is upregulated in response to DENV infection, A549 cells were infected with DENV for 72 h. Quantitative real-time PCR (qRT-PCR) analysis showed upregulation of *IRAV* starting at 24 h postinfection, corresponding to increased expression of DENV RNA, *IFN*-β, and ISGs, including *IFIT3* (Fig. 1A). To determine if IRAV is regulated through the canonical IFN (ISGF3) signaling pathway, IRF9 knockouts (KO) were generated in A549 cells using CRISPR/Cas9. Knockout of IRF9 resulted in decreased expression of IRAV after DENV infection (Fig. 1B), as well as after IFN-β treatment (Fig. 1C), indicating that the canonical ISGF3 pathway plays a role in induction of IRAV.

To further characterize IRAV induction in response to IFN, HeLa cells were treated with various concentrations of IFN-β for 16 h or were treated with IFN-β at a concentration of 3 ng/ml and collected at the indicated time points. Samples were analyzed by Western blotting for expression of IRAV and IFIT3. Analysis showed IRAV to be detectable in unstimulated HeLa cells and to be upregulated in response to treatment with IFN-β in both dose-dependent (Fig. 1D) and time-dependent (Fig. 1E) manners. To determine if IRAV was expressed in different cell lines, cells were left untreated or treated with IFN-β (10 ng/ml) for 16 h, followed by Western blot analysis for IRAV. The results showed that IRAV was upregulated in all the tested cell lines, with the exception of HEK293 cells, which produced only low levels of IRAV. In addition, it was observed that monocytes and U937 and Daudi cells produced an additional low-molecular-mass band of approximately 26 kDa that matched the predicted molecular mass of an alternate IRAV isoform (Fig. 1F).

### CRISPR-mediated knockout of IRAV results in increased titers of DENV and EMCV.

**FIG 2:**
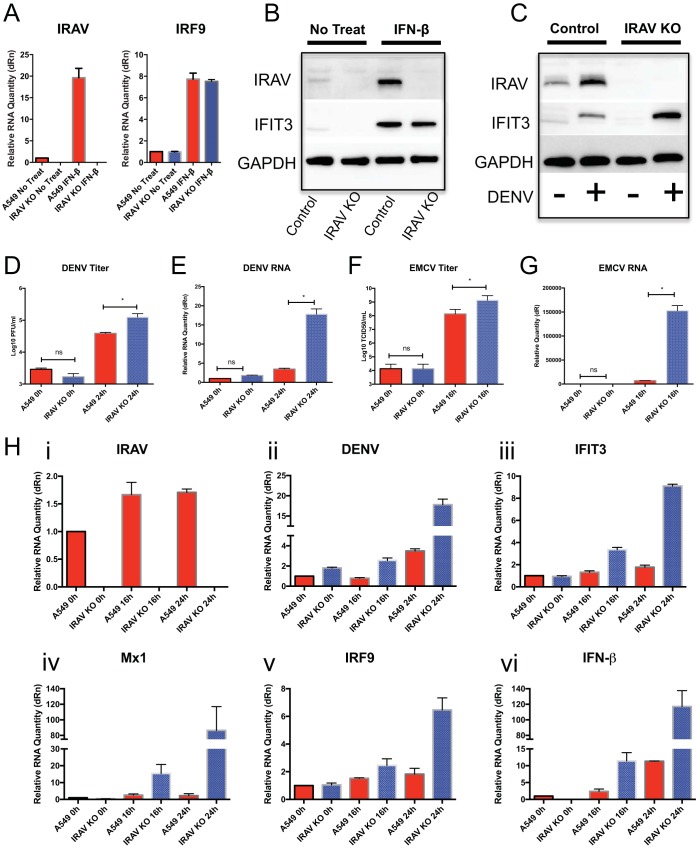
Knockout of IRAV results in enhanced replication of DENV and EMCV. (A) A549 cells or IRAV KO cells were left untreated or treated with IFN-β for 16 h, followed by qRT-PCR analysis of RNA expression of *IRAV* or *IRF9*. Samples were normalized to the HPRT housekeeping gene, and the change in expression was calculated relative to time zero. (B) Western blot analysis of A549 control or IRAV KO cells after IFN treatment. The cells were either left untreated or treated with IFN-β for 16 h, followed by Western blot analysis for IRAV or IFIT3. GAPDH was used as a loading control. (C) Western blot analysis of A549 control or IRAV KO cells after DENV infection. The cells were either left untreated (−) or infected with DENV for 72 h (+), followed by Western blotting for IRAV or IFIT3. GAPDH was used as a loading control. (D) A549 or IRAV KO cells were infected with DENV, and samples were collected at time zero and 24 h, followed by titration using the plaque assay method. (E) A549 or IRAV KO cells were infected with DENV, and samples were collected at time zero and 24 h, followed by qRT-PCR analysis of DENV RNA. (F) A549 or IRAV KO cells were infected with EMCV, and samples were collected at time zero and 16 h, followed by titration using the 50% tissue culture infective dose (TCID_50_) method. (G) A549 or IRAV KO cells were infected with EMCV, and samples were collected at time zero and 16 h, followed by qRT-PCR analysis of EMCV RNA. (H) qRT-PCR analysis of A549 or IRAV KO cells infected with DENV. Samples were collected at time zero and 16 h and 24 h postinfection and analyzed for IRAV (i) and DENV RNA (ii), as well as for expression of *IFIT3* (iii), *Mx1* (iv), *IRF9* (v), and *IFN*-β (vi). Samples were normalized to the *HPRT* housekeeping gene, and the change in expression was calculated relative to time zero. The error bars represent standard deviations. *, *P* < 0.05; ns, not significant.

The CRISPR/Cas9 system was used to generate A549 IRAV KO cells. Treatment of A549 cells with IFN-β resulted in upregulation of IRAV, as well as of other ISGs. However, in the KO cells, IRAV was not detected at either the RNA (Fig. 2A) or protein (Fig. 2B) level, while expression of other ISGs remained unaffected. Infection of A549 or IRAV KO cells with DENV (Fig. 2C) resulted in increased titers of DENV (Fig. 2D), as well as significant increases in DENV RNA relative to control cells (Fig. 2E). Similar effects were observed after infection with EMCV (Fig. 2F and G). When DENV-infected IRAV KO cells were examined for expression of other genes (Fig. 2H), we observed increased expression of the *IFIT3*, *Mx1*, *IRF9*, and *IFN*-β ISGs in IRAV KO cells relative to the control A549 cells. These results suggest that IRAV directly affects DENV replication rather than exerting its effects via regulation of other ISGs.

### IRAV associates with the dengue virus replication complex.

**FIG 3:**
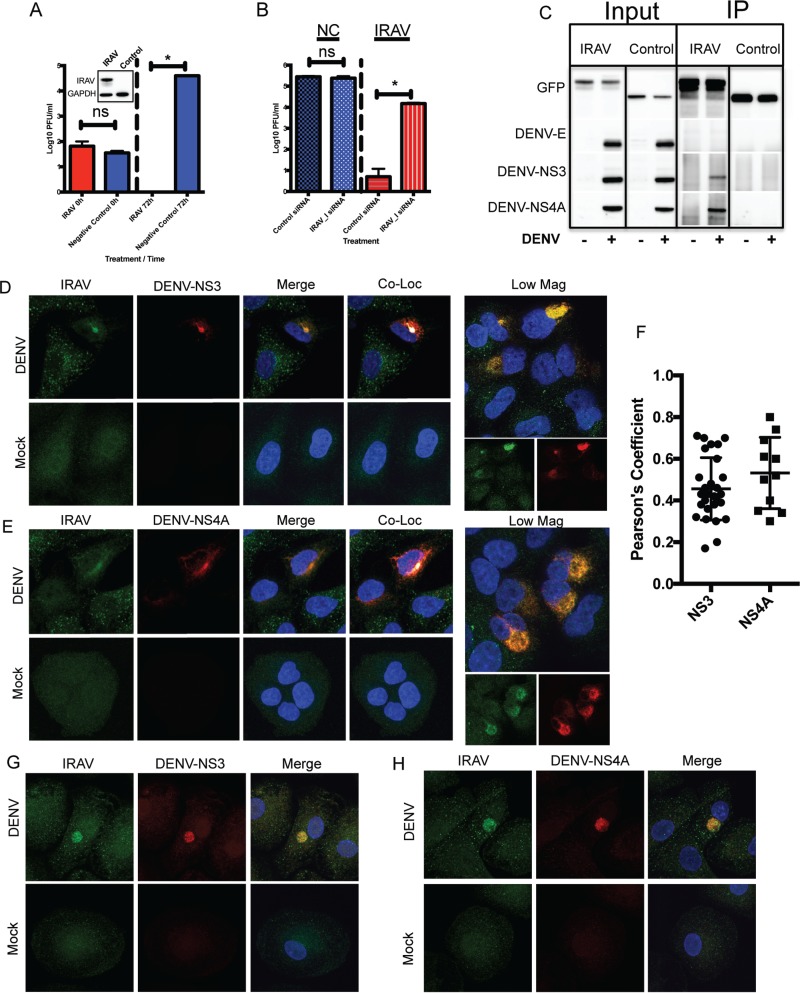
IRAV associates with the DENV replication complex. (A) HEK293 cells stably expressing IRAV or a negative control were infected with DENV at an MOI of 0.1, and samples were collected at time zero and 72 h postinfection, followed by titration using the plaque assay method. The inset represents expression of IRAV in transfected HEK293 cells compared to the negative control, as determined by Western blot analysis. GAPDH was used as a loading control. (B) HEK293 cells stably expressing IRAV or negative-control (NC) cells were treated with either negative-control siRNA or an siRNA specific to IRAV (IRAV_I). The cells were then infected with DENV for 72 h, followed by titration on Vero cells. (C) IRAV coimmunoprecipitates with DENV proteins. HEK293 cells were either left uninfected (−) or infected with DENV (+) for 48 h, followed by transfection with a plasmid expressing GFP-IRAV (IRAV) or GFP-CAT (Control) for an additional 48 h. The cell lysates were then collected, and co-IP experiments were performed using antibodies to GFP. Input and IP samples were then analyzed by Western blotting for the presence of DENV envelope (DENV-E), DENV NS3, or DENV NS4A protein. (D) Confocal microscopy of IRAV colocalized with replication complexes in DENV-infected or mock-infected A549 cells. Green, IRAV; red, DENV-NS3. The nucleus was stained with DAPI (blue). Colocalization between IRAV and DENV-NS3 is shown in white. Low Mag, a lower-magnification field showing a cluster of infected cells. (E) Confocal microscopy of IRAV colocalized with replication complexes in DENV-infected or mock-infected A549 cells. Green, IRAV; red, DENV NS4A. The nucleus was stained with DAPI (blue). Colocalization between IRAV and DENV NS4A is shown in white. (F) Colocalization coefficients of IRAV and DENV NS3 (NS3) or DENV NS4A (NS4A) in DENV-infected A549 cells as determined by Pearson's linear correlation coefficient. (G) Confocal microscopy of IRAV colocalized with replication complexes in DENV-infected or mock-infected monocyte-derived macrophages. Green, IRAV; red, DENV-NS3. The nucleus was stained with DAPI (blue). (H) Confocal microscopy of IRAV colocalized with replication complexes in DENV-infected or mock-infected monocyte-derived macrophages. Green, IRAV; red, DENV NS4A. The nucleus was stained with DAPI (blue). The error bars represent standard deviations.

To determine if IRAV has a direct influence on virus replication, HEK293 cells were stably transfected with an expression vector encoding IRAV with an amino-terminal (N-terminal) green fluorescent protein (GFP) fusion or with a negative-control vector with an N-terminal GFP tag. Because IRAV is poorly expressed in HEK293 cells (Fig. 1F), they are an ideal cell line for overexpression experiments. Cells were infected with DENV (multiplicity of infection [MOI], 0.1), and supernatants were collected at time zero and at 72 h postinfection, and the titer was determined by plaque assay. While detectable in the negative-control group, no virus was detected in cells stably expressing IRAV (Fig. 3A). The IRAV-expressing HEK293 cells were also treated with a small interfering RNA (siRNA) targeting IRAV or with a negative-control siRNA. The cells were then infected with DENV at an MOI of 0.1 for 72 h. Virus titration showed a significant increase in virus titers in IRAV-expressing HEK293 cells after knockdown of IRAV (Fig. 3B), demonstrating that IRAV has a significant influence on DENV replication even in the absence of interferon treatment. Because IRAV was previously identified by mass spectrometry (MS) as an interaction partner for HCV NS3 (34), we examined whether IRAV was interacting with DENV proteins. Coimmunoprecipitation (co-IP) experiments were performed on DENV-infected cells using GFP-IRAV as bait and GFP-chloramphenicol acetyltransferase (CAT) as a negative control. Western blotting showed that the DENV protease/helicase NS3 and the replication complex-associated protein NS4A interacted with IRAV, while the DENV envelope (E) protein did not (Fig. 3C). Interactions between IRAV and NS3 were not affected by RNase A treatment (data not shown). To further substantiate our findings, colocalization experiments were performed in A549 cells. Here, A549 cells were infected with DENV serotype 2 (DENV-2) for 48 h, followed by fixation and immunostaining for IRAV and either DENV NS3 (Fig. 3D) or NS4A (Fig. 3E) protein. While IRAV was localized to small puncta in mock-infected cells, colocalization between IRAV and NS3 or NS4A was observed in perinuclear regions of DENV-infected cells (Fig. 3F). This suggests that IRAV relocalizes to the DENV replication complex after infection. In order to demonstrate association of IRAV with the DENV replication complex in primary human cells, monocyte-derived macrophages were infected with DENV-2 as described above. As shown for A549 cells, colocalization was observed between IRAV and DENV NS3 (Fig. 3G) or NS4A (Fig. 3H) in DENV-infected monocyte-derived macrophages.

### IRAV associates with host RNA binding proteins.

**FIG 4:**
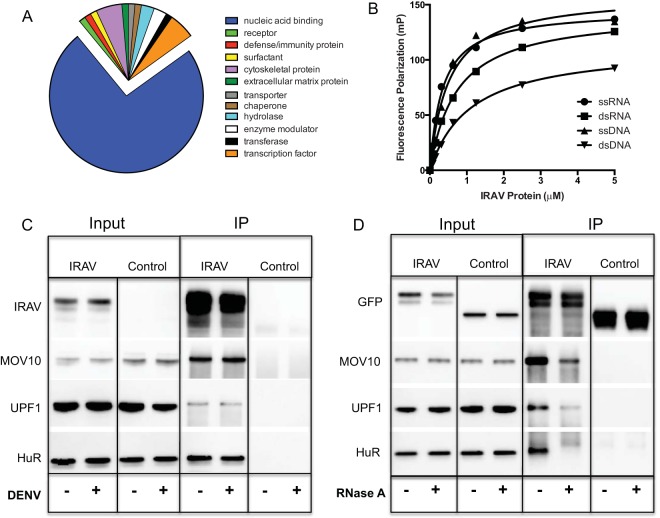
IRAV interacts with RNA binding proteins. (A) Gene ontology analysis of IRAV interaction partners as identified by co-IP of GFP-IRAV from DENV-infected HEK293 cells, followed by MS analysis. Gene ontology analysis was performed using the PANTHER classification system (http://www.pantherdb.org), based on the protein class. (B) FP assays were performed using various concentrations of recombinant IRAV and FAM-labeled 20-mer single-stranded or double-stranded DNA or RNA oligomers. Samples were run in triplicate, and FP was measured on a Hidex sense microplate reader (Turku, Finland). (C) Co-IP experiments performed on HEK293 cells left untreated (−) or infected with DENV (+). The cells were transfected with expression vectors for GFP-IRAV (IRAV) or GFP-CAT (Control), followed by IP with an antibody for GFP. Western blotting was performed for IRAV putative interaction partners MOV10, UPF1, and HuR. (D) Co-IP experiments were performed on HEK293 cells after overexpression of GFP-IRAV (IRAV) or GFP-CAT (Control). Cell lysates were either left untreated (−) or treated with RNase A (+), followed by IP with an antibody for GFP. Western blots were then performed for IRAV putative interaction partners MOV10, UPF1, and HuR.

To determine if IRAV interacted with host proteins in DENV-infected HEK293 cells, co-IP experiments were performed using overexpressed IRAV as bait. The immunoprecipitated proteins were then analyzed by MS. Gene ontology (GO) analysis was performed using the PANTHER classification system (http://pantherdb.org) (35) on MS hits with a spectral count of >50. Ontology derived based on the protein class revealed a strong preference (74%) for interactions with nucleic acid binding proteins (Fig. 4A). To characterize the nucleic acid binding properties of IRAV, fluorescence polarization (FP) assays were performed using recombinant IRAV (with an amino-terminal maltose binding protein [MBP] tag and a carboxy-terminal truncation of 18 amino acids) and synthetic 20-mer nucleic acids. Here, dilutions of IRAV were incubated with either synthetic single-stranded (ssRNA) or double-stranded (dsRNA) RNA or single- or double-stranded DNA containing complementary nucleic acid sequences. FP showed IRAV to be a nucleic acid binding protein with a higher affinity for ssRNA and ssDNA than for dsDNA (Fig. 4B).

To further explore interactions between IRAV and proteins associated with RNA processing, MS hits were ranked based on the spectral count, and the top-scoring proteins were further characterized (see Table S1 in the supplemental material). We were able to verify interactions between IRAV and MOV10, UPF1, and HuR by co-IP of overexpressed IRAV, followed by Western blotting (Fig. 4C). Because MOV10, UPF1, and HuR are all known to be RNA binding proteins, we examined the influence of RNase A treatment on these interactions. RNase treatment resulted in either complete or partial loss of interactions with IRAV (Fig. 4D). Partial loss of interactions due to RNase A treatment was previously described for interactions between MOV10 and UPF1 (36).

### IRAV colocalizes with P bodies.

**FIG 5:**
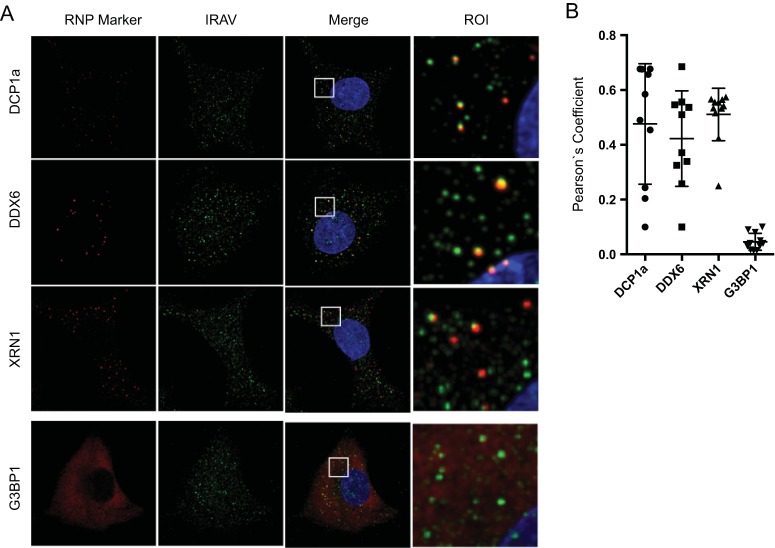
IRAV associates with P bodies in IFN-β-treated cells. (A) Confocal microscopy of IRAV; P body markers DCP1a, DDX6, and XRN1; and the stress granule marker G3BP1a in A549 cells after treatment with IFN-β (10 ng/ml) for 16 h. Green, IRAV; red, RNP markers. The nucleus was stained with DAPI (blue). Regions of interest (ROI) are boxed in white. (B) Colocalization coefficients of IRAV with DCP1a, DDX6, XRN1, and G3BP1 as determined by Pearson's linear correlation coefficient. The error bars represent standard deviations.

**FIG 6:**
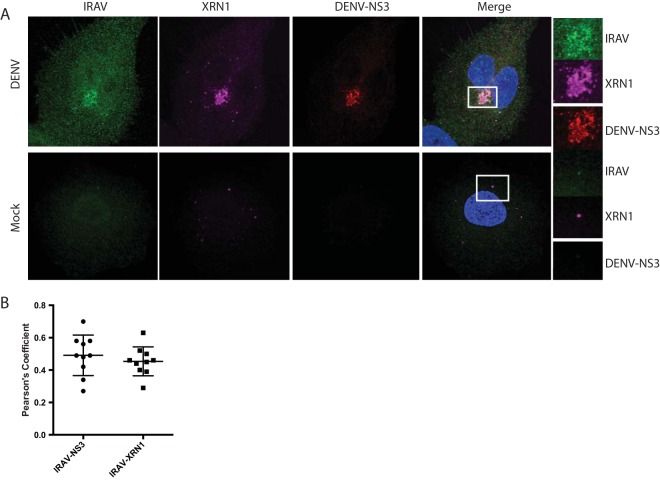
IRAV relocalizes to the replication complex after DENV infection. (A) Confocal microscopy of XRN1 colocalized with IRAV and DENV NS3 at the replication complex in DENV-infected or mock-infected A549 cells. Green, IRAV; magenta, XRN1; red, DENV NS3. The nucleus was stained with DAPI (blue). Colocalization between IRAV, XRN1, and DENV NS3 is shown in white. Regions of interest (ROI) are boxed in white. (B) Colocalization coefficients of IRAV and DENV NS3 or IRAV and XRN1 in DENV-infected A549 cells as determined by Pearson's linear correlation coefficient, demonstrating colocalization between IRAV and both XRN1 and DENV NS3 in DENV-infected cells. The error bars represent standard deviations.

Because many of the RNA binding proteins shown to interact with IRAV are associated with ribonucleoprotein (RNP) granules, we next investigated whether IRAV colocalized with specific RNP granule markers. Here, we examined A549 cells for the localization of P body markers (XRN1, DCP1a, and DDX6/RCK), as well as the stress granule marker G3BP1 (Fig. 5A). We observed colocalization between IRAV and all three P body markers in IFN-treated cells; however, no statistically significant colocalization was observed between IRAV and G3BP1, indicating that IRAV associated with P bodies in the cytoplasm of IFN-treated cells (Fig. 5B). To characterize the association of IRAV with P body components after DENV infection, A549 cells were infected with DENV, followed by staining for IRAV and XRN1 (Fig. 6A and B). As previously described for XRN1 (37), IRAV relocalized to the DENV replication complex, colocalizing with DENV NS3.

### IRAV interacts with MOV10.

**FIG 7:**
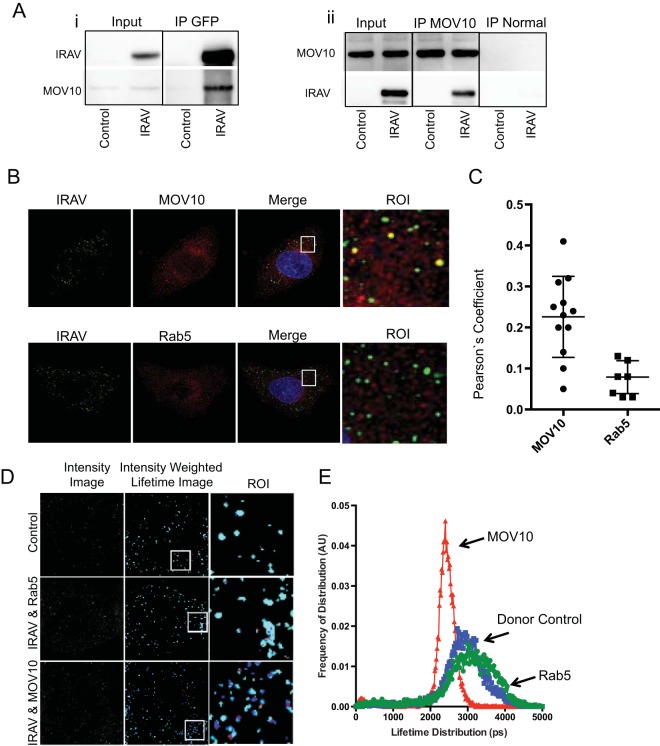
IRAV interacts with MOV10. (A) Co-IP experiments performed on HEK293 cells transfected with expression vectors for GFP-IRAV (IRAV) or GFP-CAT (Control), followed by IP with either an antibody for GFP (i) or endogenous MOV10 (ii). Normal IgG was used as a negative control for endogenous MOV10. Western blots were then performed for IRAV or MOV10. (B) Confocal microscopy of IRAV and MOV10 or Rab5 in IFN-β-treated A549 cells. Green, IRAV; red, MOV10 or Rab5. The nucleus was stained with DAPI (blue). ROI are boxed in white. (C) Colocalization coefficients of IRAV and MOV10 or Rab5 in IFN-β-treated A549 cells as determined by Pearson's linear correlation coefficient. (D) Representative image of FRET-by-FLIM analysis of IRAV interactions with MOV10 or Rab5 in IFN-β-treated A549 cells, showing the image intensity, intensity-weighted lifetime, and selected ROI. (E) Lifetime distribution plot versus frequency of distribution, in arbitrary units (AU), of FRET-by-FLIM analysis of IRAV (donor control), IRAV and MOV10, or IRAV and Rab5 in IFN-β-treated A549 cells. The plot was constructed using data from 10 images each, collected from two independent experiments. IRAV interactions with MOV10 are shown in red, Rab5 in green, and the donor control in blue. The error bars represent standard deviations.

Due to its known role as a restriction factor for retroviruses (38–41), hepatitis C virus (6), and hepatitis B virus (42), we further characterized interactions between MOV10 and IRAV. Co-IPs and reciprocal co-IPs were performed using HEK293 cells transfected with either IRAV (GFP-IRAV) or a negative control (GFP-CAT). Co-IPs were then performed using an antibody either to overexpressed IRAV or to endogenous MOV10, and Western blots were analyzed for the presence of either MOV10 or IRAV (Fig. 7A). In addition, confocal microscopy and fluorescence resonance energy transfer (FRET) by fluorescence lifetime imaging (FLIM) analyses were used to characterize interactions between endogenous IRAV and MOV10 in A549 cells. Confocal microscopy revealed colocalization between IRAV and MOV10, as determined by Pearson's linear correlation coefficient (Fig. 7B and C). Moreover, FRET-by-FLIM analysis demonstrated interactions between IRAV and MOV10, with lifetime values of 1.8 to 2.1 compared to values of 2.5 to 2.8 for the donor control, representing 25% to 28% FRET efficiency. The endosomal marker Rab5 was used as a negative control. Rab5 did not demonstrate significant colocalization with IRAV or a shift in lifetime values (Fig. 7D and E).

**FIG 8:**
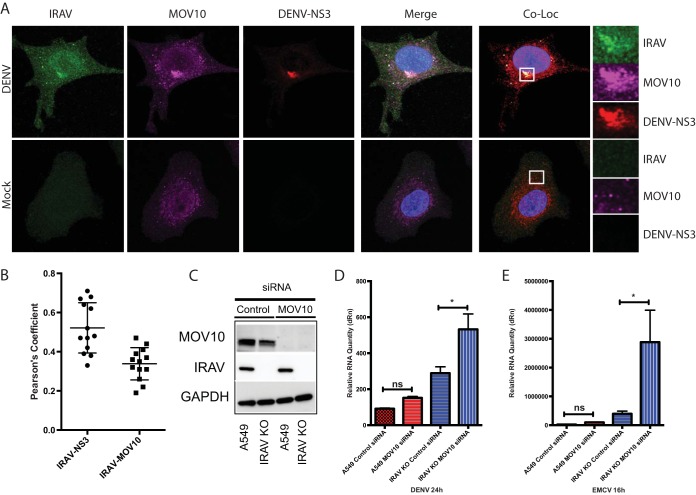
MOV10 localizes to the DENV replication complex and restricts viral replication. (A) Confocal microscopy of MOV10 colocalized with IRAV and DENV NS3 at the replication complex in DENV-infected or mock-infected A549 cells. Green, IRAV; magenta, MOV10; red, DENV NS3. The nucleus was stained with DAPI (blue). Colocalization between IRAV, MOV10, and DENV NS3 is shown in white. ROI are boxed in white. (B) Colocalization coefficients of IRAV and DENV NS3 or IRAV and MOV10 in DENV-infected A549 cells as determined by Pearson's linear correlation coefficient, demonstrating colocalization between IRAV and both MOV10 and DENV NS3. (C) Western blot analysis of siRNA-mediated knockdown of MOV10 in A549 or IRAV KO cells. The cells were treated with a gene-specific siRNA for MOV10 (Qiagen Hs_MOV10_5 FlexiTube siRNA) or a negative-control siRNA for 48 h, followed by Western blot analysis for MOV10 or IRAV. GAPDH was used as a loading control. (D) qRT-PCR analysis of A549 or IRAV KO cells treated with either negative-control siRNA or gene-specific MOV10 siRNA for 48 h. The cells were then infected with DENV (MOI, 0.1) for 24 h and analyzed for DENV RNA. Samples were normalized to the HPRT housekeeping gene, and the change in expression was calculated relative to time zero. (E) qRT-PCR analysis of A549 or IRAV KO cells treated with either negative-control siRNA or gene-specific MOV10 siRNA for 48 h. The cells were then infected with EMCV (MOI, 0.001) for 24 h and analyzed for EMCV RNA. Samples were normalized to the HPRT housekeeping gene, and the change in expression was calculated relative to time zero. The error bars represent standard deviations. *, *P* < 0.05; ns, not significant.

Confocal microscopy was also used to examine localization of IRAV and MOV10 in DENV-infected and mock-infected A549 cells (Fig. 8A and B). Here, it was observed that, similar to XRN1, both IRAV and MOV10 relocalized to the viral replication complex, colocalizing with DENV NS3. To further substantiate a role for MOV10 in restriction of DENV replication, siRNA-mediated knockdown experiments were performed in A549 and IRAV KO cells (Fig. 8C). Depletion of MOV10 resulted in an increase in virus replication for DENV, as well as for EMCV, which was significantly greater in IRAV KO cells than in IRAV-expressing A549 cells (Fig. 8D and E), indicating that both IRAV and MOV10 play roles in the restriction of virus replication.

## DISCUSSION

Interferon-stimulated genes are key mediators of antiviral immunity and play a pivotal role in the immune response to DENV infection. While the IFN response is an innate and broad-spectrum response to many pathogens, ISGs have been demonstrated to have remarkable specificity both for the viruses they target and the pathways through which they inhibit viral replication (6). Hundreds of ISGs have been shown to be upregulated in response to induction of IFN signaling pathways, illustrating the scope and complexity of the IFN response. To date, only a fraction of these genes have been characterized.

Here, we show that IRAV is an IFN-stimulated gene with antiviral activity against DENV. Knockout of the IFN signaling component IRF9 results in a significant reduction of IRAV expression after IFN treatment, suggesting regulation through the canonical ISGF3 complex; however, it should be noted that there appears to be some baseline constitutive expression of IRAV. In addition, IRAV homologues were identified in hemichordates and echinoderms, organisms that lack a “classical” IFN response, suggesting the possibility of IFN-independent functions for IRAV-like proteins.

IRAV associates with P bodies, as evidenced by interactions with P body-associated proteins (MOV10 and UPF1) and colocalization with P body markers (DCP1a, XRN1, and DDX6). P bodies are discrete cytoplasmic structures composed of RNA and proteins that are associated with various aspects of RNA turnover, including nonsense-mediated decay, adenylate-uridylate-rich element (ARE)-mediated decay, and gene silencing (43). Notably, both DENV and West Nile virus (WNV) have been shown to associate with components of P bodies. Subgenomic flavivirus RNA (sfRNA) inhibits the activity of XRN1 and TRIM25 (44–46), and DDX6 interacts with DENV untranslated regions (UTRs) and localizes to DENV replication complexes, possibly playing a role in virus replication (47). Furthermore, WNV infection of HeLa cells results in a decrease of both the size and number of P bodies, along with a relocalization of P body components (including GW182, DDX3, and XRN1) to WNV replication complexes, colocalizing with NS3 (37). This is similar to what we observed with IRAV during DENV infection, with IRAV puncta becoming more diffuse and colocalizing with NS3 in distinct perinuclear complexes. A number of other viruses, including hepatitis C virus (48), poliovirus (49), influenza virus (50), and bunyaviruses (51, 52), have been shown to interact with P bodies or with P body components (53).

MOV10 and UPF1 are both members of the SF1 family of helicases and have been previously shown to interact with one another and to form complexes with APOBEC3G and Argonaute 2 (40, 54), as well as with the antiviral protein ZAP (55). Interactions between MOV10 and UPF1 are partially sensitive to RNase treatment (36), as we have also demonstrated for IRAV interactions with MOV10 or UPF1. Knockdown of MOV10 results in an increase in the half-lives of mRNA targets (36). MOV10 has been shown to be involved in retrotransposition of LINE elements (56–58), as well as in microRNA pathways (54, 59). MOV10 may also play a role in mediating antiviral response (60) and restricts replication of retroviruses (38–41), hepatitis C virus (6), hepatitis B virus (42), and influenza virus (61). UPF1 is a key component of nonsense-mediated RNA decay pathways (62) and has been demonstrated to play a role in viral replication cycles (63, 64). IRAV also associates with the RNA binding protein HuR. HuR has been shown to be involved in RNA decay pathways (65) and has been linked to viral replication and modulation of the host response (66–69). In addition, IRAV has been previously shown to interact with RNA binding proteins LARP1 and PABPC1, both of which have also been shown to be involved in RNA decay (23). Taken together, IRAV's role as an RNA binding protein, its association with proteins linked to RNA decay, and its localization to P bodies (sites of RNA decay) and the marked increase in viral RNA observed after knockout of IRAV all suggest a role for IRAV in degradation of viral RNA. The role of MOV10 in IRAV-mediated antiviral activity remains unclear; however, given MOV10's role as an RNA helicase involved in RNA decay pathways, as well as its localization at the viral replication complex and the increase in viral RNA observed after its knockdown, MOV10 may function in conjunction with IRAV and other proteins to aid in destabilization of viral RNA.

In conclusion, we show that *IRAV* is an ISG that is regulated through the canonical type I interferon signaling pathway. IRAV displays antiviral activity against DENV and EMCV and interacts with DENV proteins. IRAV is an RNA binding protein that localizes to P bodies, sites of RNA decay. Additionally, IRAV interacts with MOV10 and UPF1, two proteins previously shown to interact with each other and to be involved in RNA decay pathways. These data serve to identify an ISG with antiviral activity against DENV, as well as to suggest a mechanism of action involving the destabilization of viral RNA.

## MATERIALS AND METHODS

### Viruses and cell culture.

The DENV serotype 2 isolate Tonga/74 was generously provided by S. Whitehead (Laboratory of Infectious Diseases [LID], NIAID) and passaged in C6/36 cells. The titer of virus was determined on Vero cells as previously described (70, 71). EMCV and vesicular stomatitis virus (VSV) were obtained from the American Type Culture Collection (ATCC) (Manassas, VA) and passaged in Vero cells. Virus infections were performed at the indicated MOI, and virus was allowed to adsorb to the cells for 1 hour. Unbound virus was then removed, and the cells were washed with phosphate-buffered saline (PBS), followed by the addition of RPMI medium containing 10% fetal bovine serum. Primary human monocytes were obtained using the Gambro Elutra method from the NIH Clinical Research Center Department of Transfusion. Monocyte-derived macrophages were cultured as previously described (72). HEK293 cells were obtained from Life Technologies (Grand Island, NY). Daudi cells (a human B lymphoblast line) were obtained from P. Grimley (Department of Pathology, Uniformed Services University of the Health Sciences, Bethesda, MD). The human epithelial breast adenocarcinoma cell line MDA-MB-231 was obtained from Raj Puri (FDA, Center for Biologics Evaluation and Research [CBER], Bethesda, MD), OVCAR3 (human ovarian adenocarcinoma) cells were obtained from the National Cancer Institute (Bethesda, MD). Human cervical carcinoma HeLa S3 cells (CCL-2.2); a human monocyte-like cell line derived from histiocytic lymphoma (U937 [CRL-1593.2]); and Detroit 551 (CCL-110), NCI-H1299 (CRL-5803), HEL 299 (CCL-137), and human lung carcinoma A549 (CCL-185) cell lines were obtained from the ATCC. All the cell lines were cultured in RPMI medium containing 10% fetal bovine serum and 50 μg/ml of penicillin and streptomycin. IFN-β1a (Avonex), with a specific activity of 200 million IU per mg, was obtained from Biogen Idec (Cambridge, MA).

### Antibodies and fluorescent labeling.

Primary antibodies were obtained as follows: C19orf66 (FLJ11286; HPA042001), Sigma-Aldrich Corp., St. Louis, MO; UPF1 (D15G6), Cell Signaling Technology, Inc., Danvers, MA; Rab5 (C8B1), Cell Signaling Technology, Inc., Danvers, MA; GAPDH (glyceraldehyde-3-phosphate dehydrogenase) (D16H11), Cell Signaling Technology, Inc., Danvers, MA; MOV10 (A301-571A), Bethyl Laboratories, Inc., Montgomery, TX; XRN1 (C-1), Santa Cruz Biotechnology, Inc., Dallas, TX; HuR (G-8), Santa Cruz Biotechnology, Inc., Dallas, TX; hDcp1a (65-Y), Santa Cruz Biotechnology, Inc., Dallas, TX; RCK (DDX6; E-12), Santa Cruz Biotechnology, Inc., Dallas, TX; G3BP1 (H-10), Santa Cruz Biotechnology, Inc., Dallas, TX; IRF9 (ISGF3γ; 610285), BD Biosciences, San Jose, CA; IFIT3, Covance, Inc., Princeton, NJ; NS4A (SAB2700179), Sigma-Aldrich Corp., St. Louis, MO; NS3 (SAB2700181), Sigma-Aldrich Corp., St. Louis, MO; and E (SAB2700196), Sigma-Aldrich Corp., St. Louis, MO.

Horseradish peroxidase (HRP)-labeled secondary antibodies for Western blotting were obtained from Santa Cruz Biotechnology, Inc. Fluorescently labeled secondary antibodies for confocal microscopy were obtained from Life Technologies (Grand Island, NY). Fluorescent labeling of primary antibodies for colocalization and FRET-by-FLIM of IRAV and MOV10, Rab5, DENV NS3, and DENV NS4A were performed using Zenon antibody-labeling kits (Life Technologies) according to the manufacturer's specifications.

### Plasmid constructs and transfections.

A plasmid containing the IRAV gene was purchased from GeneCopeia (Rockville, MD) and amplified using directional forward and reverse primers (5′-CAC CAT GTC TCA GGA AGG TGT GGA-3′ and 5′-GGC GGG CCC AGG GAG TGA-3′), followed by cloning into a pENTR/D-TOPO vector (Life Technologies, Grand Island, NY). The IRAV gene sequence was then transferred to a pcDNA 6.2/N-EmGFP-DEST vector (Life Technologies) via LR recombination using Gateway LR Clonase enzyme mix (Life Technologies) according to the manufacturer's protocol. The constructs were sequenced to verify correct sequence and orientation. Negative-control vectors were obtained from Life Technologies. Transfections were performed in HEK293 cells using Opti-MEM and X-tremeGene HP according to the manufacturer's directions (Roche Diagnostics, Indianapolis, IN).

### CRISPR/Cas9 knockout and siRNA knockdown experiments.

CRISPR/Cas9 knockout cell lines were generated using A549 cells. An FLJ11286/C19orf66 (IRAV) CRISPR/Cas9 knockout plasmid (sc-408037), containing the Cas9 nuclease and guide RNA, and its complementary homology-directed repair plasmid (sc-408037), containing red fluorescent protein and a puromycin resistance gene flanked by 5′ and 3′ arms homologous to regions flanking the Cas9 target site, or an IRF9 CRISPR/Cas9 knockout plasmid (sc-400958) and a homology-directed repair plasmid (sc-400958), were obtained from Santa Cruz Biotechnology, Inc. (Dallas, TX). A549 cells were cotransfected with CRISPR/Cas9 and homology-directed repair plasmids using GenMute transfection reagent (SignaGen Laboratories, Ijamsville, MD), and knockouts were screened for puromycin resistance (3 μg/μl). Individual subclones were then assayed for gene expression by both PCR and Western blotting. Knockdown experiments were performed using gene-specific siRNAs. IRAV siRNA (Hs_FLJ11286_5; catalog number S104203668), MOV10 siRNA (Hs_MOV10_5 FlexiTube siRNA), and AllStars negative-control siRNA (SI03650318) were obtained from Qiagen (Valencia, CA). Reverse transfections were performed using Lipofectamine RNAiMax transfection reagent (Life Technologies), according to the manufacturer's specifications.

### Immunoprecipitation, mass spectrometry, and Western blot analysis.

Protein coimmunoprecipitations of GFP-IRAV- or GFP-CAT-transfected cells and Western blot analyses were performed as previously described (73). Identification of gel-separated proteins was performed on reduced and alkylated, trypsin-digested samples. The supernatant and two washes (5% formic acid in 50% acetonitrile) of the gel digests were pooled and concentrated by speed vac (Labconco, Kansas, MO) to dryness directly in 200-μl polypropylene autosampler vials (Sun Sri, Rockwood, TN). The recovered peptides were resuspended in 5 μl of solvent A (0.1% formic acid, 2% acetonitrile, and 97.9% water). Prior to mass spectrometry analysis, the resuspended peptides were chromatographed directly on column, without trap cleanup. The bound peptides were separated at 500 nl/min, generating 80 × 10^5^ to 120 × 10^5^ Pa pressure, using an AQ C_18_ reverse-phase medium (3-μm particle size and 200-μm pore size) packed in a pulled-tip nanochromatography column (0.100-mm inside diameter [i.d.] by 150-mm length) from Precision Capillary Columns (San Clemente, CA). The chromatography was performed in line with an LTQ-Velos Orbitrap mass spectrometer (ThermoFisher Scientific, West Palm Beach, FL), and the mobile phase consisted of a linear gradient prepared from solvent A and solvent B (0.1% formic acid, 2% water, and 97.9% acetonitrile) at room temperature. Nano-liquid chromatography (LC)-MS (LC–tandem MS [MS-MS]) was performed with a ProXeon Easy-nLC II multidimensional liquid chromatograph and a temperature-controlled Ion Max Nanospray source (ThermoFisher Scientific) in line with the LTQ-Velos Orbitrap mass spectrometer.

A Mascot (Matrix Science, Beachwood, OH) search of the data was performed against a concatenated sequence file containing dengue virus proteins found in the UniProtKB TrEMBL database, human protein UniProtKB Swiss-Prot, and the common contaminant proteins found in the GPM.org's cRAP database, all downloaded in March 2014. The data were searched with 2 allowed missed cleavages and mass tolerances of 15 ppm and 0.8 Da for the precursor and fragment ions, respectively. Carbamidomethylation of cysteine was set as a fixed modification, while oxidation of methionine and deamidation of asparagine and glutamine were searched as dynamic modifications. The resulting search files were reclustered against the same sequence database for further analysis using ProteoIQ software (Premier Biosoft, Palo Alto, CA). Filters of 2 spectra per peptide sequence and 2 peptides per protein were in place for protein assignments. The data were then ranked based on the number of peptide hits and the spectral count.

### Quantitative real-time PCRs.

RNA was extracted from DENV-infected cells using a Qiagen RNeasy minikit and QIAshredder spin columns and including DNase digestion (Qiagen, Valencia, CA). qRT-PCRs were performed using the SensiFast Probe No-ROX One-Step kit (Bioline USA Inc., Taunton, MA), and reactions were run on a Stratagene Mx3005P real-time thermocycler (Agilent Technologies, Santa Clara, CA). Primers and probes were obtained from Integrated DNA Technologies, Inc. (Coralville, IA), and were as follows: DENV-2 (forward, 5′-TGC CTA CAG TTC TAC GTC TCC-3′; reverse, 5′-TCG TTT CCT AAC AAT CCC ACC-3′; probe, 5′-6-carboxyfluorescein (FAM)-CCT TCC AAT-ZEN-CTC TTT CCT GAA GCC TCT C-Iowa black fluorescence quencher [IABkFQ]-3′), EMCV (forward, 5′-TTC AGC GTT TTC TAC TCC CTG-3′; reverse, 5′-TCA CTC CCC TCA CTT ACC C-3′; probe, 5′-FAM-AGA AAT CCT-ZEN-TCC CTG CGC TCA CC-IABkFQ-3′), IFN-β (forward, 5′-TGA AGC AAT TGT CCA GTC CC-3′; reverse, 5′-GCC AAG GAG TAC AGT CAC TG-3′; probe, 5′-FAM-AGG CAC AGG-ZEN-CTA GGA GAT CTT CAG T-IABkFQ-3′), IRAV (forward, 5′-CTA AGT AAC GAT CTG GAT GCC C-3′; reverse, 5′-CGT TGA AAC ATG CGT AGG TTG-3′; probe, 5′-hexachlorofluorescein [HEX]-CCT GAA TGT-ZEN-CCC GGT CCT GCT T-IABkFQ-3′), HPRT (forward, 5′-GTA TTC ATT ATA GTC AAG GGC ATA TCC-3′; reverse, 5′-AGA TGG TCA AGG TCG CAA G-3′; probe, 5′-Cy5-TGG TGA AAA GGA CCC CAC GAA GT-Iowa black RQ-Sp [IAbRQSp]-3′), IRF9 (forward, 5′-CAA AGG CCT GCT CCA TCT-3′; reverse, 5′-TGC AGA GAC TTG GTC AGG TA-3′; probe, 5′-FAM-CAT GGC TCT-ZEN-CTT CCC AGA AAT TCA GTG T-IABkFQ-3′), MX1 (forward, 5′-CCA CCC ATA TTT CAG GGA TCT G-3′; reverse, 5′-TCT GGT GAG TCT CCT TGA TTT G-3′; probe, 5′-HEX-TGT GTG ATG AGC TCG CTG GTA AGT TT-IABkFQ-3′), and IFIT3 (forward, 5′-CAC TGT CTT CCT TGA ATA AGT TCC-3′; reverse, 5′-AGA ACA AAT CAG CCT GGT CAC-3′; probe, 5′-HEX-AGA AAA TCC-ZEN-TTC CAC AGC TGA AAT GCC-IABkFQ-3′).

### Protein expression, purification, and fluorescence polarization.

The nucleotide sequence representing the first 269 aa of IRAV was amplified by PCR using forward and reverse primers (5′-CTA GTC GAC ATG TCT CAG GAA GGT GTG GAG CTG GAG-3′ and 5′-CTA GCG GCC GCC AGG ATG AGG TTG TCC AGG TCT TCC AGG AGG-3′) and ligated into a modified MBP-tagged expression vector with a pET30a backbone. The BL-21-CodonPlus RIPL strain (Stratagene, Santa Clara, CA) was then transformed with the plasmid, and recombinant protein was induced with 0.2 mM IPTG (isopropyl-β-d-thiogalactopyranoside) at 18°C for 4 h. The soluble fraction of the recombinant protein was purified by Ni-nitrilotriacetic acid (NTA) chromatography, followed by Superdex-200 gel filtration with 200 mM NaCl, 20 mM HEPES, pH 7.4. The nonaggregate fraction of the His-MBP-tagged IRAV protein was analyzed by SDS-PAGE as ∼95% pure.

For FP assays, the following FAM-labeled 30-mer RNA probes were used: 5′-rArGrU rUrGrU rUrArG rUrCrU rArCrG rUrGrG rArC/36-FAM-3′, with or without the reverse complement 5′-rGrUrC rCrArC rGrUrA rGrArC rUrArA rCrArA rCrU-3′ (where “r” refers to ribose); or DNA probes 5′-AGT TGT TAG TCT ACG TGG AC-FAM-3′, with or without the reverse complement 5′-GTC CAC GTA GAC TAA CAA CT-3′.

Oligomers were dissolved in binding buffer (100 mM KCl, 20 mM HEPES, pH 7.4), heated to 85°C for 10 min, and allowed to cool slowly. The oligomers were then added to serial dilutions of protein solution in triplicate, and FP was measured on a Corning black 96-well, half-area assay plate (Corning, NY) using a Hidex Sense Microplate Reader (Turku, Finland).

### Confocal microscopy and fluorescence resonance energy transfer by fluorescence lifetime imaging.

A549 cells were plated on poly-d-lysine-coated 35-mm culture dishes (MatTek, Ashland, MA) and either treated with IFN-β (10 ng/ml) for 16 h or infected with DENV-2 (MOI, 3) for 48 h. The cells were fixed with 4% paraformaldehyde in PBS and permeabilized with 0.1% or 0.5% Triton X-100, followed by blocking with 5% bovine serum albumin (BSA) for 30 min. Primary antibodies were diluted in 10% normal goat serum and incubated at room temperature for 2 h, followed by three washes in PBS and staining with fluorescently labeled secondary antibody (1:500) and nuclear DAPI (4′,6-diamidino-2-phenylindole) stain (Life Technologies). Primary labeled antibodies were diluted in 10% normal goat serum and incubated at room temperature for 2 h, followed by three washes in PBS, and postfixed with 4% paraformaldehyde in PBS. Images were collected on a Leica SP8 inverted confocal microscope with a 63× oil immersion objective (Leica Microsystems, Buffalo Grove, IL). Colocalization analysis was performed using Imaris software (Bitplane Inc., South Windsor, CT). FRET-by-FLIM analysis was performed as previously described (73).

### Gene ontology and statistical analyses.

GO analysis was performed using proteins identified by MS with spectral counts above 50. Selected protein accessions were analyzed using the PANTHER classification system (http://pantherdb.org) (35). All statistical analyses were performed on Prism (GraphPad Software, Inc.), using one-way analysis of variance (ANOVA) with Tukey's *post hoc* test and a *P* value of 0.05.

## Supplementary Material

Supplemental material
