# Supplementary material for: IRAV (FLJ11286), an Interferon-Stimulated Gene with Antiviral Activity against Dengue Virus, Interacts with MOV10
Source: J Virol. 2017 Feb 14;91(5):e01606-16. doi: 10.1128/JVI.01606-16 (PMC5309953; doi:10.1128/JVI.01606-16)
Supplement: Supplemental material [file supp_91_5_e01606-16__index.html]

IRAV (FLJ11286), an Interferon-Stimulated Gene with Antiviral Activity against Dengue Virus, Interacts with MOV10 — Supplemental material 

# *IRAV* (*FLJ11286*), an Interferon-Stimulated Gene with Antiviral Activity against Dengue Virus, Interacts with MOV10

## Supplemental material

- Supplemental file 1 -

  Table S1 (IRAV interaction partners identified by MS.)

  PDF, 93K
