# Supplementary material for: IRAV (FLJ11286), an Interferon-Stimulated Gene with Antiviral Activity against Dengue Virus, Interacts with MOV10
Source: J Virol. 2017 Feb 14;91(5):e01606-16. doi: 10.1128/JVI.01606-16 (PMC5309953; doi:10.1128/JVI.01606-16)
Supplement: Supplemental material [file JVI.01606-16_zjv999182364s1.pdf]

Table S1. IRAV Interaction Partners Identified by MS.

Number	Sequence Id	Sequence Name	Protein Length (aa)	Peptides	% Sequence Coverage	Spectral Count
**1**	**Q9NUL5**	**FLJ11286 (IRAV)**	**291**	**25**	**75.95**	**6756**
2	Q6PKG0	LARP1	1096	56	60.49	1813
3	Q14980	*NUMA1*	2115	79	47.04	1280
4	P43243	*MATR3*	847	35	46.87	1226
5	Q09161	NCBP1	790	30	49.11	984
6	P67809	YBX1	324	15	53.7	900
7	Q14152	*EIF3A*	1382	49	36.25	837
8	Q92900	UPF1 (RENT1)	1129	41	45.44	582
9	P62249	*RPS16*	146	13	63.7	571
10	Q9HCE1	MOV10	1003	36	40.08	454
11	P16989	YBX3	372	13	35.75	437
12	Q15717	HuR (ELAVL1)	326	15	53.37	429
13	P62701	*RPS4X*	263	14	49.05	424
14	Q15149	*PLEC*	4684	72	16.52	409
15	P22626	*HNRNPA2B1*	353	17	50.71	367
16	Q04637	*EIF4G1*	1599	28	24.64	349
17	P09651	*HNRNPA1*	372	14	44.89	325
18	P23246	*SFPQ*	707	19	35.36	312
19	Q9H307	PNN	717	14	22.87	299
20	P52597	*HNRNPF*	415	11	45.54	269
21	Q9Y520	PRRC2C	2896	27	11.57	268
22	P23588	*EIF4B*	611	17	32.41	265
23	Q3KQU3	MAP7D1	841	18	25.21	247
24	Q99613	*EIF3C*	913	10	14.35	236
25	P15924	*DSP*	2871	38	14.63	231
26	Q9NZB2	FAM120A	1118	17	19.32	218
27	Q9BXP5	SRRT	876	16	25.23	211
28	P49756	RBM25	843	17	28.94	202

MS analysis of IRAV interaction partners identified by Co-IP. Table is representative of MS hits ranked based on spectral count (>200).
